# Family caregivers’ perceptions of quality of dementia self‐care in Wakiso district, Uganda

**DOI:** 10.1002/alz.70510

**Published:** 2025-07-19

**Authors:** Dennis Rogers Buwembo, Juliet Kiguli, Noeline Nakasujja, Fredrick Edward Makumbi, Martha Sajatovic, Kylie Meyer, Kamada Lwere, Joy Louise Gumikirza‐Onoria, Joseph Kagaayi, William Ddaaki, Mark Kaddu Mukasa

**Affiliations:** ^1^ Brain Health Training Program School of Medicine College of Health Sciences Makerere University Kampala Uganda; ^2^ Department of Community Health and Behavioral Sciences School of Public Health College of Health Sciences Makerere University Kampala Uganda; ^3^ Department of Psychiatry School of Medicine College of Health Sciences Makerere University Kampala Uganda; ^4^ Department of Epidemiology and Biostatistics School of Public Health College of Health Sciences Makerere University Kampala Uganda; ^5^ Neurological and Behavioral Outcomes Center School of Medicine University Hospitals Cleveland Medical Center Case Western Reserve University Cleveland Ohio USA; ^6^ Frances Payne Bolton School of Nursing Case Western Reserve University Cleveland Ohio USA; ^7^ Department of Microbiology School of Biomedical Sciences College of Health Sciences Makerere University Kampala Uganda; ^8^ Social and Behavioral Sciences Research Rakai Health Sciences Program Kyotera Uganda; ^9^ Department of Internal Medicine School of Medicine College of Health Sciences Makerere University Kampala Uganda

## Abstract

**INTRODUCTION:**

In Sub‐Saharan Africa, most people with dementia are cared for in the home setting (dementia self‐care) with hardly any support from the formal health and social care system. The study explored family caregivers' perceptions of quality of dementia self‐care.

**METHODS:**

A descriptive‐exploratory qualitative study design was used. Data were collected through four focus group discussions with 48 family caregivers of older people with dementia, recruited from four villages of the Wakiso district in Uganda.

**RESULTS:**

Five themes emerged: (1) patience and understanding; (2) maintaining hygiene and cleanliness; (3) constant supervision and safety precautions; (4) personalized care by understanding individual preferences, daily routines, and personal space; (5) respect and dignity.

**DISCUSSION:**

Family caregivers’ perceptions of quality of dementia self‐care are generally positive, encompassing elements of person‐centered care. Caregiver messages should be strengthened by incorporating support with instrumental activities of daily living and how to avoid harmful or potentially harmful caregiver behaviors.

**Highlights:**

- Family caregivers’ perceptions of dementia self‐care are critical, especially in contexts in which knowledge is limited, and myths, misconceptions, and stigma prevail.
- Caregivers’ views emphasize the importance of valuing care recipients, understanding their preferences, and tailoring care accordingly.
- These perceptions prioritize ensuring safety and supporting basic daily activities, with minimal attention to instrumental activities.
- Caregivers hardly reflected on their behaviors that could potentially harm the care recipients.

## 1 BACKGROUND

In low‐ and middle‐income countries (LMICs) like Uganda, dementia care is predominantly informal, relying on unpaid, untrained caregivers, typically family members.^1^, ^2^ Nearly all (96%) of people with dementia live at home,^3^ receiving care from family members with or without support from formal health and social services. We use the term dementia self‐care instead of informal care to align with the World Health Organization's (WHO) definition of self‐care. WHO defines self‐care as the ability of individuals, families, and communities to promote health, prevent disease, maintain health, and cope with illness and disability with or without the support of a health worker.^4^ Despite this dominant role of family caregiving, by untrained, unprepared, and unpaid family caregivers, little is known about how family caregivers perceive the quality of the care they provide. Understanding how family caregivers perceive quality of dementia self‐care is crucial, as these perceptions are vital to achieving the goal of dementia care, which is “living well with dementia”^5^ in such countries.

Dementia self‐care involves various tasks, including supporting basic and instrumental daily living activities, addressing emotional needs, managing behavioral symptoms, and arranging health and social care visits. Family caregivers also navigate complex challenges, including safeguarding the rights of the older person with dementia within their home environment.^6^ As dementia progresses, caregiving tasks become more difficult,^1^ requiring increased effort from family caregivers. In LMICs, particularly in Sub‐Saharan Africa, dementia caregiving is of particular concern because support from the formal health and social care systems is largely absent,^2^ yet, recent projections indicate that the majority of the estimated 139 million people that will be living with dementia worldwide by 2050 will reside in LMICs.^5^

The costs of dementia caregiving are significant. In 2015, family caregivers contributed ≈ 82 billion hours of care, with 60.9% of this time spent by family caregivers in LMICs. Notably, 69% of total dementia care costs in this setting stem from informal caregiving.^3^ The burden of caregiving disproportionately falls on female family members—spouses, daughters, and daughters‐in‐law^3^, ^6^, ^7^—who often have minimal formal training, are unprepared, and unpaid for the demands of the role. In addition to the lack of training, caregivers in these settings operate in a context of low dementia awareness, stigma, and misconceptions. Dementia is often wrongly attributed to aging, witchcraft, or divine punishment, resulting in discrimination and sometimes even abuse of the older person with dementia, particularly when their behavior is perceived as socially inappropriate. ^8^, ^9^

Most research on dementia caregiving is from high‐income countries, focusing largely on the caregiving burden. Literature on experiences of caregiving reports how cultural factors influence caregiving quality. In particular, caregivers’ attitudes and beliefs toward meeting the emotional and physical needs of the older person with dementia, while balancing their own needs, are shaped by cultural beliefs.^10^, ^11^, ^12^ Other factors such as caregivers’ knowledge of dementia, employment status, relationship to the older person with dementia (with spouses generally assuming greater responsibility), and sex (with women typically experiencing more emotional burden) also affect caregiving quality.^13^, ^14^, ^15^, ^16^, ^17^, ^18^, ^19^ Existing research on dementia caregiving in Sub‐Saharan Africa is limited, but, to a large extent it collaborates that from high‐income countries.^9^, ^20^, ^21^, ^22^, ^23^ However, this research was conducted in a few countries, mostly South Africa,^9^ making it difficult to generalize findings to the broader African context. The diverse cultural, linguistic, and caregiving environments across the region may influence caregiving practices in unique ways. Additionally, demographic shifts—such as declining fertility rates, changing generational attitudes, rural‐to‐urban migration, and expanded educational opportunities for women—will likely impact the future of family caregiving.^1^

Given these complexities, exploring family caregivers’ perceptions of dementia self‐care quality is crucial for developing interventions that can improve caregivers’ experiences and outcomes for people with dementia. Understanding these perceptions will help enhance the well‐being of individuals living with dementia in Uganda and similar contexts within Sub‐Saharan Africa.

## 2 METHODS

### 2.1 Study design

We used an exploratory‐descriptive qualitative research design^24^ to investigate the perceptions of family caregivers of people living with dementia regarding the quality of dementia self‐care. This approach is grounded in the understanding that there is no absolute or universal understanding of what quality in dementia self‐care is. Instead, family caregivers construct meaning by interpreting and making sense of their caregiving experiences within their socio‐cultural contexts.

**RESEARCH IN CONTEXT**

1. **Systematic review**: Quality of dementia care in the home setting has not been comprehensively conceptualized or studied. Therefore, our aim was to map currently available literature on the topic. We used scoping review methods to search PubMed and Google Scholar for literature on informal care for older people with dementia of any type. Relevant studies identified were summarized and cited appropriately.
2. **Interpretation**: Our findings show that family caregivers’ perceptions of dementia care in the home settings are positively aligned with person‐centered care, and focus almost exclusively on ensuring safety and support with basic activities of daily living.
3. **Future directions**: We propose what needs to be included in family caregivers’ messages or trainings aimed at improving quality of dementia care in the home setting. Future research should test the effects of these messages/trainings on quality of care provided by family caregivers.

### 2.2 Context/study setting

The study was conducted in a community‐based setting in Wakiso District, Uganda.

We purposively selected villages from both Nansana municipality and Busukuma division to ensure a diverse representation of both urban and rural^25^ study participants, capturing a wide range of caregiving experiences from both contexts. Specifically, four villages: Nabitalo‐Kiwenda, Kabumbi‐Nansana, Kayebe‐Gayaza, and Kibuuku‐Busukuma were selected. Wakiso is the most populous district in Uganda, with a population of 3,397,565 people^26^ comprising indigenous people and settlers from all over the country. The majority of people are of low social economic status either employed in informal settings such as subsistence farming, casual laborers, and market vending and so on, or unemployed.^25^

### 2.3 Study participants

Given the reported low levels of male participation in dementia caregiving research,^14^ we aimed to capture perspectives and experiences of both women and men across a range of ages from both rural and urban contexts. We used maximum variation purposeful/purposive sampling^27^, ^28^, ^29^ to select a diverse group of primary caregivers of older individuals with dementia. Study participants were chosen based on key dimensions such as age, sex, and rural or urban residence. This approach was taken to make sure that the study results in an accurate and comprehensive description of central themes common to all participants, regardless of their specific characteristics.^27^ Caregivers were recruited with the help of a village health team (VHT) member who knew people that were caring for those diagnosed with dementia in their setting. To be eligible to participate in the study, caregivers had to be aged ≥ 18 years, or an emancipated minor, and the primary caregiver to an older adult (aged ≥ 60 years) diagnosed with dementia. Caregivers were excluded from the study if they had any health condition that could impede their full and active participation.

### 2.4 Ethical issues/clearance

The study was cleared by the higher degrees committee of the School of Public Health, College of Health Sciences, and received ethical clearance from the Makerere School of Public Health, Research and Ethics Committee (MaKSPHREC), study registration number SPH‐2022‐354.

### 2.5 Data collection procedures

Data were collected from October 16 to 27, 2023, through four focus group discussions (FGDs) with 12 participants per group. All FGD participants provided written informed consent. Data saturation was deemed achieved when no new information emerged from the discussions.^30^ The FGDs were moderated by a clinical psychologist (J.L.G.‐O.) who has experience in qualitative research methods, and assisted by a public health specialist (D.R.B.) serving as the note taker. The discussions were guided by an open‐ended interview guide, which included the following questions:

1. What does quality of dementia self‐care mean to you?
2. How should older people with dementia be cared for?
3. What constitutes good‐ and poor‐quality dementia self‐care?
4. What are the benefits of providing good‐ or poor‐quality dementia self‐care?
5. What are some of the reasons for not meeting dementia self‐care needs?

The FGDs were conducted in Luganda, the most commonly spoken language in the Wakiso district, to ensure that participants’ opinions and nuances were fully understood. Sessions lasted between 88 minutes (1 hour 28 minutes) to 2 hours. All discussions were audio recorded and subsequently transcribed verbatim into English. Two of the authors (D.R.B. and W.D.), carefully reviewed the translated transcripts against the audio recordings and the field notes to ensure transcription accuracy and the preservation of meaning prior to data analysis.

### 2.6 Data analysis

We used a six‐step framework^31^ to conduct a structured thematic analysis and identify recurrent patterns in transcripts of FGDs on dementia caregiving. The analysis was carried out jointly by two researchers: D.R.B., a public health specialist with medical training in dementia care and W.D., a social scientist with limited dementia‐specific knowledge, but extensive experience in qualitative data analysis. Initial informal and unfocused data analysis^27^ began during data collection. Data saturation was judged to have been reached by the fourth FGD, when participants’ responses began to noticeably resemble those from the previous FGD.^30^ Formal and focused data analysis^27^ commenced with both analysts independently reviewing the transcripts, FGD recordings, and field notes. Participant responses were organized according to the FGD guide questions. NVivo 12 software was used to facilitate data coding and theme development.

Through an inductive approach, the analysts, worked independently but collaboratively over a period of 3 months, developed a consensus‐based codebook comprising 37 codes. These codes reflected the participants’ meanings in response to the FGD questions. The codes were applied to the entire dataset^32^ enabling the identification of nine preliminary themes: (1) what constitutes good care; (2) understanding care, which was broken into (a) dimensions of care and (b) perceptions of care; (3) outcomes associated with good care; (4) constraints to maintaining optimal care; (5) effects of good caregiving on the caregiver; (6) expectations and preferences in caregiving; (7) factors influencing caregiving gaps; (8) indicators of sub‐optimal care and potential risks; and (9) immediate and long‐term consequences of poor care.

After a critical review of the core themes for coherence and distinctiveness, three core themes were retained. Subsequently, through a deductive and iterative process, guided by existing dementia caregiving literature, five final themes were identified. This refinement involved the addition, merging, and reclassification of codes and aligning them with established conceptual frameworks.^27^, ^28^, ^29^ For example, the theme “constant supervision and safety” emerged from reframing codes initially categorized under “what constitutes good care.”

### 2.7 Techniques to enhance trustworthiness

We relied on techniques described to bolster trustworthiness of our research findings.^33^ The FGD moderator ensured the establishment of trust and rapport with participants by thoroughly explaining the study's goal, objectives, and procedures. Participants were assured that their individual responses would remain confidential, and it was emphasized that their participation was entirely voluntary. They were also informed that they could withdraw from the study at any point if they felt uncomfortable, ensuring they could openly share their perspectives. We did not perform the formal member‐checking because of resource constraints, but the moderator paraphrased each participant's response to confirm the intended meaning had been captured.^34^ Additionally, a detailed description of both the participants and the study context is provided, enabling readers to assess the transferability of the findings to their own settings.

## 3 RESULTS

Forty‐eight participants agreed to participate in the study; details are included in Table 1. Most (> 80%) of the study participants were females with ages ranging from 18 years to 63 years.

**TABLE 1:** Composition of focus group discussions.

Focus group	Sex composition		Age range	Context
	Females	Males		
1	11	01	19–60	Rural
2	07	05	18–35	Urban
3	12	0	22–52	Urban
4	10	02	25‐63	Rural

Five themes emerged from the FGDs: (1) patience and understanding; (2) maintaining hygiene and cleanliness; (3) constant supervision and safety precautions; (4) personalized care by understanding individual preferences, daily routines, and personal space; and (5) respect and dignity. Each will be discussed in the sections that follow.

### 3.1 Patience and understanding

Caregivers reported that caring for an older person with dementia is a challenging undertaking. Because of the symptoms associated with dementia such as forgetfulness, confusion, and irritability, caregivers need to be empathetic rather than responding with frustration. Caregivers reported that older people with dementia often resist care, yet they need it. Because older people with dementia have other co‐morbidities, medication must be taken on time, but sometimes they refuse. Due to challenges with memory, one minute the older person with dementia is requesting for something, the next they are questioning why you are doing something for them. Oftentimes they are stuck in the past with both good and bad memories and therefore their mood is constantly changing. These behaviors are challenging to the caregivers who unfortunately must remain calm to contain the situation, because if the caregiver gets annoyed, scolding older people with dementia kills their morale and makes them withdraw.

“My grandmother also has diabetes, the health workers advised us to give her medication at certain time intervals, however, she sometimes refuses to take the medicine as scheduled. At times she can express desire to eat something and when you bring it, she rejects it and asks for something completely different” (Female caregiver #08, FGD2).

“Grandmother lost all her children in a road accident, while they were traveling to celebrate Christmas with her. But, every year around 20th December, she starts preparing for her children's visit by fetching firewood, collecting bunches of banana, and so on, while doing this she is in high spirits, then the next morning she goes to the grave yard to clean around the graves and she will be sad the whole day” (Female caregiver #01, FGD4).

### 3.2 Maintaining hygiene and cleanliness

Caregivers perceived older people with dementia to be in need of support, which involves but is not limited to keeping their living spaces clean and tidy, including their bedrooms, bathrooms, and common areas. Additionally, caregivers mentioned that it was important to make sure that the older person with dementia's personal hygiene is maintained through provision of assistance with bathing, changing clothes, and toileting. Caregivers believed that the immunity of older people with dementia is low and therefore they are at increased risk of infections especially if left uncared for after they have soiled or wetted their clothes. Not only are the older people with dementia unable to take care of themselves with regard to basic activities of daily living (ADLs), caregivers reported that older people with dementia oftentimes soil their clothes, therefore it is incumbent upon the caregiver to make sure that they maintain their hygiene appropriately so that they don't get infections, which could worsen their illness.

“Care recipients are old people who can get diseases easily, therefore, as the caregiver I need to make sure that my mother lives in a clean environment, everything I do should be done in a clean way” (Female caregiver #010, FGD1).

“So, you need to be there and come to their aid, by cleaning after them. For some of these people we bathe them, wash their clothes using detergents, ensure they change into clean clothes and then you clean up after what they have wetted or soiled” (Female caregiver #07, FGD2).

### 3.3 Constant supervision and safety

Caregivers noted that older people with dementia often get confused, and are unaware of the surroundings and place. As such they mostly collide into obstacles in their paths and fall easily. Sometimes they get lost as they frequently wander off into the neighborhood. Because of the above, to make sure that older people with dementia are safe, caregivers have to pay close attention so that older people with dementia don't hurt themselves or get lost. Dangerous objects need to be kept out of their reach and one needs to constantly monitor their whereabouts to prevent them from wandering off unsupervised.

“My grandmother behaves in a confused way, you can find her fallen on the ground and when you ask her where she has been going, she tells you that she was going to the kitchen, yet she is in the direction of the toilets. The time she got lost, she said she was going to visit the neighbor, but she was in a different direction” (Female caregiver #06, FGD4).

“In my experience, the old man I care for is forgetful most of the time. Therefore, I can't allow him to get out of my sight, because, if you are with him and you walk off to attend to something else, by the time you come back, he is gone because our home doesn't have a fence. So, you have to keep him in sight twenty‐four seven” (Male caregiver #09, FGD2).

### 3.4 Personalized care by understanding individual preferences, daily routines, and personal space

Over the years older people form their routines, including choices and preferences for what they want to do at certain times as well as likes and dislikes for certain foods. Caregivers reported that older people with dementia often become sad when the caregiver acts contrary to their choices and preferences. Caregivers noted that older people with dementia have individualized needs, which require close interaction and engagement to be communicated and well understood.

“I later realized that grandmother dislikes beans and silver fish, whenever I served her beans or silver fish, she would ignore the food, insisting that she will eat it later, only to abandon it altogether and go without food the whole day” (Female caregiver #05, FGD1).

“The quality of care means the way I treat the older person with dementia, I ask him about his favorite music and when I play the music and I request him to dance for us, while he is dancing, we cheer him on while clapping” (Male caregiver #11 FGD3).

### 3.5 Respect and dignity

Caregivers reported that older people with dementia should be treated with respect and dignity by speaking to them in a kind and respectful manner, even when they are agitated or confused. Caregivers expressed a need to show compassion, understanding, and willingness to engage with and include the older person with dementia in decision making. Caregivers believed that older people with dementia need regular interaction and meaningful engagement with those around them, to foster a trusting relationship. Meaningful connections created through activities such as engaging in talks/discussions about past events, activities that they once enjoyed—music, dancing, cooking, and so on, were thought to be a determinant of quality of care, because they foster creation of an atmosphere of friendship and trust, which allows the older person with dementia to express their preferences and concerns freely. Engaging older people with dementia in conversations, actively listening to and involving them in decision making about daily home activities greatly enhances their sense of agency and dignity.

“Grandmother refuses to eat the same food repeatedly no matter how you plead. So, I engage her to find out if she remembers what she ate the previous day, if she doesn't remember I remind her and seek for her opinion on what we should eat today” (Female caregiver #11, FGD2).

“You have to respect them, despite their current situation. It is like when they soil (defecate/urinate) on themselves, you have to look after (clean) them well and calmly tell them not to do it again. They are like children; you don't have to scold them” (Female caregiver #04 FGD1).

## 4 DISCUSSION

This study, conducted in the Wakiso district of Uganda, explored the perceptions of family caregivers of older people with dementia regarding the quality of dementia self‐care. Across caregiving contexts (rural or urban), sex, and age, caregivers believed that older people with dementia require support with mostly basic ADLs in a manner that recognizes their preferences and needs, while also treating them with respect and dignity. An additional key concern was the need for constant monitoring and supervision of older people with dementia to prevent harm from accidents or incidents of wandering, a common behavior among older people with dementia.

The findings of this study build on previous research by demonstrating that family caregivers’ responsibilities extend beyond providing assistance with ADLs. Caregivers also attend to the emotional and psychological needs of the older person with dementia.^35^, ^36^, ^37^ It has been emphasized in the literature that care for individuals with dementia should be person‐centered, prioritizing mindfulness and respect for the older person with dementia's individual needs, preferences, identity, and social status.^1^ Treating older people with dementia respectfully is in line with cultural norms in Uganda. Traditionally, elders are regarded as a source of knowledge and wealth. They play an advisory role of guiding and providing for the upbringing of children and grandchildren as well as mediating family/community conflicts.^38^ The respect afforded to older people in general is crucial in promoting caregivers’ ability to respond to challenging behaviors with patience, empathy, and compassion—traits commonly associated with high‐quality dementia care.^36^

Caregivers in the study emphasized their role in providing more support for basic activities such as bathing, dressing, cleaning, and so on, compared to instrumental activities such as managing finances, answering phone calls, and so on, of daily living, with a strong focus on ensuring the safety of the older person with dementia through continuous monitoring. This may suggest that dementia in Uganda is often diagnosed at an advanced stage of the illness, as previous studies have reported dementia patients to have similar symptoms such as forgetfulness, incontinence, and wandering among individuals with dementia.^23^ This proactive attention to safety is commendable, as the caregiving burden has been shown to increase alongside the growing need for support with ADLs and safety concerns.^39^

Caregivers predominantly highlighted actions reflective of good‐quality dementia self‐care. This selective reporting might suggest a tendency to underplay negative aspects of caregiving, possibly due to social desirability bias.^40^ Nonetheless, the provision of high‐quality dementia self‐care may be more prevalent in this setting due to several factors that reduce the caregiving burden and enhance the caregiver's ability to cope. These factors may include spiritual, religious, and cultural values attached to caregiving, the sharing of caregiving responsibilities, the caregiver residing in the same household as the older person with dementia, and the presence of larger households^9^—typical of Ugandan family settings, in which the average household size is 4.4 people.^26^

### 4.1 Strength and limitations

This study explored perceptions of caregivers’ of older people with dementia from both urban and rural areas in Uganda, ensuring a diverse range of views. Additionally, a concerted effort was made to include male caregivers, who have historically been underrepresented in dementia caregiving studies. We conducted four FGDs involving 48 caregivers of older people with dementia, 8 of whom were male. This approach enabled us to capture perceptions from a heterogeneous sample of caregivers. However, there is a possibility that caregivers may have censored certain information or selectively shared views they deemed socially acceptable in the group setting. We mitigated this risk by having an experienced clinical psychologist moderate the FGDs. To encourage open and honest discussions, the moderator assured the study participants that their responses would not be directly connected to their names, thus protecting their confidentiality.

## 5 CONCLUSION

This study contributes to the literature on dementia self‐care by highlighting the perceptions of caregivers of older people with dementia in Uganda, particularly in terms of the quality of dementia self‐care. Across caregiving contexts (rural and urban), age, and sex, caregivers demonstrated a strong understanding of the need for respect, empathy, and individualized care. Additionally, they noted that challenges associated with caregiving for older people with dementia, such as safety concerns and emotional support, remain critical. Caregivers’ messages on quality of dementia self‐care should include support with instrumental ADLs and potentially or harmful caregivers’ behaviors. Future research should further explore how caregivers can be better supported to provide high‐quality care, particularly in low‐resource settings like Uganda.

## CONFLICT OF INTEREST STATEMENT

All authors declare no competing conflicts of interest. Author disclosures are available in the supporting information.

## CONSENT STATEMENT

All human subjects provided informed consent to participate in this study.

## Supporting information



Supporting Information
